# Response of under-ground bud bank to degradation in an alpine meadows on the Qinghai-Tibet Plateau, China

**DOI:** 10.3389/fpls.2022.1013331

**Published:** 2022-11-01

**Authors:** Jun Yang, Min Zhang, Xiang-tao Wang

**Affiliations:** ^1^ Chongqing Institute of Green and Intelligent Technology, Chinese Academy of Sciences, Chongqing, China; ^2^ University of Chinese Academy of Sciences, Beijing, China; ^3^ College of Animal Science, Tibet Agricultural and Animal Husbandry University, Nyingchi, China; ^4^ College of Life Science, China West Normal University, Nanchong, China; ^5^ Qiangtang Alpine Grassland Ecosystem Research Station (jointly built with Lanzhou University), Tibet Agricultural and Animal Husbandry University, Nyingchi, China; ^6^ Collaborative Innovation Centre of Ecological Grassland Animal Husbandry in Tibet Plateau, Nyingchi, China

**Keywords:** bud bank, vegetation restoration, degradation, vegetative reproduction, alpine meadow

## Abstract

Exploring the diversity and formation mechanism of under-ground bud banks is essential for understanding the renewal of plant populations and community succession. However, there are few studies on the response of bud bank size and composition to different degradation gradients in alpine meadows. In view of this, we investigated the size and composition of bud bank under four degradation gradients (non-degraded:ND, lightly degraded:LD, moderately degraded:MD, and heavily degraded:HD) caused by overgrazing in a typical alpine meadow in Tibet, China, using a unit area excavation sampling method, and analyzed the correlation between above-ground plant community composition and bud bank density. Our results showed that: (i) in the ND alpine meadow, rhizome buds were dominant, in the LD, tiller buds were dominant, and in the MD, root-sprouting buds were dominant; (ii) total bud bank and cyperaceae bud density decreased with increasing degradation gradient, the density of leguminosae was insignificant in each degradation gradient, and the density of gramineae and forb were dominant in LD and MD meadows, respectively; (iii) total bud bank density was significantly and positively correlated with total above-ground biomass in the LD gradient, tiller bud density was significantly positively correlated with the species diversity index of above-ground vegetation under the ND gradient, rhizome bud density was significantly and positively correlated with total above-ground biomass in the LD gradient, and root-sprouting density was significantly negatively correlated with total above-ground biomass in ND meadows, but was significantly positively correlated with the species diversity index of the LD gradient. Therefore, our research shows that rhizome buds are more important in ND meadow habitats, tiller buds are more important in LD meadow habitats, and root-sprouting buds are more important in MD meadows. The response of bud banks to degradation gradient varies with different types of bud banks and different functional groups of plants, and the survival strategy of bud banks is of great value for community restoration and regeneration, which should be paid more attention to in subsequent alpine meadow research.

## Introduction

Grassland ecosystems account for about one-third of the earth’s coverage area, provide habitats for various fauna and flora, and play an important role in the maintenance of ecosystem services such as climate regulation, water conservation, and support for humans (Curtin and Western, 2008; Ott et al., 2019). However, as global climate change and human activities intensify, abiotic limiting factors (moisture, temperature, pH, etc.) affecting vegetation growth change, especially in arid or semi-arid grassland ecosystems, and irrational land use practices and overgrazing are considered to be the main threats to grassland degradation (Bai et al., 2007; Min et al., 2011; Zwicke et al., 2013; Yin et al., 2013). In the grassland ecosystems, the vegetation reproduction bank is composed of an asexually cloned bud bank and a sexually reproduced seed bank (Abernethy and Willby, 1999; Klimešová et al., 2017). In grassland ecosystems dominated by annuals, the maintenance of grassland plant communities and the protection of genetic variants depend on seed banks, and the under-ground seed bank is highly similar to above-ground communities, but in perennial grassland ecosystems dominated by clonal propagation, the composition of grassland vegetation, seasonal changes and the spatial and temporal patterns of vegetation productivity depend on the bud bank (Gough, 2006; Klimeš, 2008; Silvertown et al., 1993; Vítová et al., 2017).

The type of bud represents the way in which the plant is derived, and the bud bank is divided into different types based on the different taxonomic categories and the location of the rooted buds. In grassland ecosystems, most new bud recruitment in perennial herbaceous plants occurs from under-ground axillary buds (rhizome buds or tiller buds) (Ott and Hartnett, 2012), with a small proportion coming from adventitious root buds. The response of herbaceous communities to environmental disturbances depends not only on the number of buds, but also on the type of bud bank and vegetative functional groups (Aarssen, 2008; Qian et al., 2017). For example, *Leymus chinensis* produces more rhizome buds under high water content conditions and more tiller buds under low water content (Wang et al., 2008). The reduction in rhizome buds and the increase in tiller buds of vegetation under high temperature stress greatly contribute to the growth of net primary productivity above-ground (Camilla and Kevin, 2014). In resource-rich ecosystems, plants can have multiple types of buds at the same time, including tiller buds, scale buds and rhizome buds, while under soil-poor conditions plants grow mainly rhizome buds (Klimešová and Klimeš, 2008; Rusch et al., 2011). In addition, grazing increased the branch number and the density of bud banks of cyperaceae, compared to the density of bud banks and branch number of gramineae, which were not affected (Damhoureyeh and Hartnett, 1997).

In recent decades, re-vegetation and sand control in alpine meadow ecosystems have gradually become a hot topic of research (Jackson et al., 1985; Hoover et al., 2014; Lin et al., 2016; Song et al., 2020; Wang F et al., 2020). In alpine meadows, stresses and disturbances coexist, with climate change and overgrazing being the main factors in meadow degradation (Wessels et al., 2007; Rutherford et al., 2011), both of which alter soil environmental conditions (e.g. soil temperature, moisture, nutrients, etc.) for plant growth (Xiao et al., 2015). For example, degraded meadows become more susceptible to rainfall erosion and weathering, which reduces the soil’s ability to store water and fertiliser, leading to increased spatial heterogeneity between vegetation and soil, which in turn affects vegetation growth, community succession and ecosystem function (Krzic and Bomke et al., 2000; Zhou et al., 2012). In addition, palatability is an important factor for livestock to selectively feed on forage (Zheng et al., 2006). In this process, livestock tend to choose high protein, good palatability forage and not to feed on low protein, poor palatability forage or to feed less on it. This means that the selective foraging of grassland plants by livestock results in a significant substitution change in the dominance of the major species in the community, with herbivores in tall-grass prairie releasing weeds that are in competitive suppression by C4 plants, leading to an increase in community diversity and species richness of forb (Towne et al., 2005). In addition to this, the mechanical activity of large hoofed herbivores under overgrazing conditions will increase soil compaction and limit seed dispersal and germination capacity (Klimešová et al., 2009; Herben et al., 2016). Unlike sexual reproduction, which relies on seed propagation, under-ground bud banks have direct access to the resources of the parent plant and rely on its high survival rate and strong reproductive capacity for rapid growth to restore populations (Dong, 2011).

The alpine meadow is the largest and most widely distributed typical alpine grassland ecosystem in China, covering an area of about 1.2 × 10^6^ km^2^, accounting for about 47.05% of the total area of the Qinghai-Tibet Plateau (Tian et al., 2014). In the past time, due to overgrazing and irrational use of land, the alpine meadows in this area have been degraded to varying degrees, which severely restricted the ecosystem service functions of alpine meadows. As the type and density of under-ground bud banks are important mechanisms for resisting disturbances in grassland degradation and maintaining stable plant populations, it is essential to explore the role of different bud bank types and densities in population regeneration and re-vegetation under degradation gradients.

This study investigated the dynamics of three bud bank types in alpine meadows under four meadow degradation types (the non-degraded, lightly degraded, moderately degraded and heavily degraded meadow), with the aim of determining the response of bud banks to degradation gradients and the relationship with above-ground vegetation composition in Tibetan alpine meadows in China. We addressed three questions: (1) How do subsurface bud bank densities and types respond to disturbances in degradation gradients? (2) How does the response of bud banks to degradation gradients differ between meadow functional groups? (3) What is the relationship between bud bank density and above-ground plant communities under different degradation gradients? Comparing the basic ecological strategies of bud banks under degradation gradients will help to predict vegetation succession and maintain the rational use of grasslands.

## Materials and methods

### Study site

**Figure 1:**
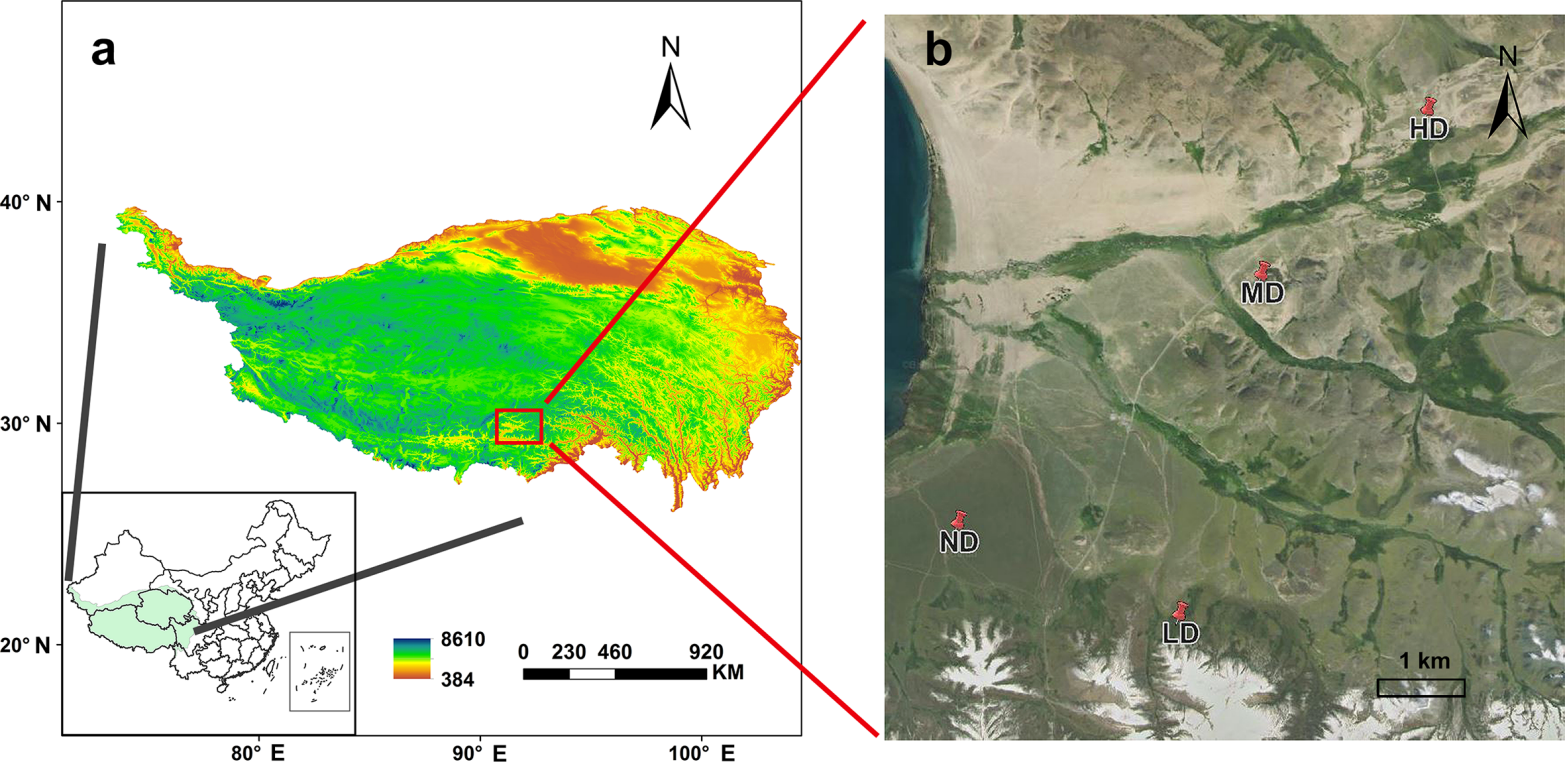
The study area-locates near the Damxung Grassland Station (30°51′N, 91°05′E, 4333 m a.s.l) on the Qinghai-Tibet Plateau **(A)**. non-degraded alpine meadow (ND); lightly-degraded alpine meadow (LD); moderately degraded alpine meadow (MD); heavily degraded alpine meadow Figure 1(HD) **(B)**.

This study was conducted near the Damxung Grassland Station (30°51′N, 91°05′E, 4333 m a.s.l), on the southern slope of the Nyenchen Tanglha in Qinghai-Tibet Plateau (**Figure 1A**). The region belongs to the plateau continental climate. The mean annual precipitation is 459.6 mm, of which about 80% occurs during the growing season (May to September). The mean annual temperature is 1.6°C, ranging from -10°C in January to 12.5°C in June (Zhang et al., 2009). There were no significant differences in temperature and precipitation in the sampling year compared to the history mean. The grassland is a typical alpine meadow vegetation type, the main plants are *Kobresia pygmaea*, *Kobresia humilis* and *Stipa capillacea*.

### Evaluation of soil degradation

An evaluation system based on vegetation survey was established near the experimental station to evaluate the degradation status of the meadow (Wen et al., 2010; Yang et al., 2020; Wang et al., 2021), which was composed of vegetation composition, ground fragmentation degree (the degree of surface fragmentation is the degree of fragmentation of the alpine meadow surface felt layer, expressed as the percentage of intact felt layer per unit area), coverage degree and plant biomass, and the study site was divided into four degraded gradient meadows (**Table 1** **;** **Figure 1B**). Specifically, the ground surface of the non-degraded meadow (ND) was almost unbroken, and the dominant species was *Kobresia pygmaea* with vegetation coverage greater than 80%; The degree of ground surface fragmentation was obvious in the lightly degraded meadow (LD), and the dominant species were *Stipa capillacea* and *Carex montis-everestii*, the number of forb such as *Potentilla saundersiana* increased significantly, and the vegetation coverage was 60%-80%; In the moderately degraded meadow (MD), the surface fragmentation was more serious, the felts were scattered on the surface, and the dominant position of forb such as *Pleurospermum hedinii* and *Leontopodium nanum* was more obvious, and the vegetation coverage was 40%-60%; The heavily degraded meadow (HD) surface basically bare, the felt disappeared, and the vegetation coverage was less than 40%, forb such as *Leontopodium nanum* and *Artemisia wellbyi* occupy the dominant position. Fences were set in the four sample plots to allow seasonal grazing (the plants were not affected by livestock and human activities in the growing season, and livestock were allowed to eat in the non-growing season).

**Table 1:** Geographical information and species composition of the study sites.

Degradationlevel	Latitude(N)	Longitude(E)	Altitude(m)	Richnessindex	Shannonindex	Biomass(g/m^2^)	Dominant Species
ND	30°C45′ 19′′	91°C03′ 23′′	4456	8 ± 1b	1.23 ± 0.05c	1502.6 ± 8.7a	*Stipa capillacea*, *Stipa purpurea*, *Kobresia pygmaea*
LD	30°C43′ 48′′	91°C07′ 41′′	4389	7 ± 1b	1.50 ± 0.11b	1070.9 ± 7.5b	*Stipa capillacea*, *Carex montis-everestii*
MD	30°C49′ 31′′	91°C09′ 17′′	4350	12 ± 1a	1.78 ± 0.06a	1203.0 ± 16.7b	*Pleurospermum hedinii*, *Leontopodium nanum*, *Artemisia wellbyi*
HD	30°C52′ 15′′	91°C12′ 32′′	4348	8 ± 1b	1.77 ± 0.10a	431.7 ± 13.2c	*Leontopodium nanum*, *Artemisia wellbyi*

Different lowercase letters in the same column indicate significant differences under different degradation gradients (P < 0.05), Data are means ± standard error. non-degraded alpine meadow (ND); lightly-degraded alpine meadow (LD); moderately degraded alpine meadow (MD); heavily degraded alpine meadow (HD). The error bars represent the standard error of the mean (n = 3).

Different lowercase letters in the same column indicate significant differences under different degradation gradients (*P* < 0.05), Data are means ± standard error. non-degraded alpine meadow (ND); lightly-degraded alpine meadow (LD); moderately degraded alpine meadow (MD); heavily degraded alpine meadow (HD). The error bars represent the standard error of the mean (n = 3).

### Community and bud bank sampling

In August 2018, plant community and bud bank surveys were conducted by digging out the entire soil core in four selected degraded meadows (ND, LD, MD, HD). The entire experimental area covered about 100 hm^2^, with each degraded meadow measuring approximately 15-20 hm^2^, five plots of 100 × 100 m were randomly assigned to each degraded meadow, each plot is more than 500 m apart, five small quadrats (0.5 × 0.5 m) were randomly set for each plot to investigate plant species, height and coverage (relevant results can be found in previous studies at this sample site) (Yang et al., 2020). After the plant community survey, the quadrats were excavated to a depth of 0.5 m, the soil cores were shaken off and the above-ground and below-ground parts of the plants were kept intact (to facilitate species identification and bud counts) and placed in a ziplock bag to be taken back to the laboratory. All species occurred in the sampling sites and the study sites had species that could reproduce both by seed and bud bank, such as the *Stipa purpurea*.

### Sample processing

We took the plants back to the laboratory, washed them with clean water, and then identified the species, bud bank type and number. In this case, the Latin names of the plant species were took from the online website: Flora of China (http://www.iplant.cn/). During the identification process, only intact bud tissue was recorded, and any necrotic bud tissue was discarded. According to the criteria (survival strategy and response phenotype of the bud bank) proposed by Ma (Ott and Hartnett, 2012; Ma et al., 2019), the under-ground bud bank (buds growing in soil) was divided into three types: rhizome buds (buds are buds that grow on the rhizomes of plants and can be counted directly), tiller buds (buds formed mainly at the tiller bases of gramineae and need to be counted after dissecting the base of the tillering node), root-sprouting bud (buds are adventitious buds that grow on the roots of plants and can be counted directly), and different plant functional groups (cyperaceae, gramineae, leguminosae, forb) for classification and identification (**Table 2**). Each species was classified and identified by naked eye or under anatomic microscope in combination with morphological characteristics of buds and attached organ parts.

**Table 2:** Species list and their bud bank types in the study region.

Plant functional group	Species	Bud bank type
cyperaceae	*Kobresia pygmaea*, *Kobresia humilis*, *Carex montiseverestii*	rhizome
gramineae	*Stipa capillacea*, *Stipa purpurea*, *Poa litwinowiana*, *Festuca coelestis *	tiller
leguminosae	*Astragalus strictus*, *Astragalus confertus*, *Astragalus tribulifolius*	root-sprouting
forb	*Youngia simulatrix*, *Artemisia wellbyi*, *Heteropappus bowerii*, *Pleurospermum hedinii*, *Potentilla cuneata*, *Stellera chamaejasme*, *Potentilla saundersiana*, *Leontopodium nanum*, *Potentilla bifurca*, *Anaphalis xylorhiza *	root-sprouting

### Data analysis

The original data of bud density was converted to the standard number of buds per m^2^. We first used a Kolmogorov-Smirnov (K-S) test to examined whether the data were normally distributed. One-way analysis of variance (ANOVA) was applied to analyze (1) the response of bud bank density to four degraded alpine meadows, (2) the difference in bud bank density of three types of bud bank, and (3) the difference of the bud bank types of the four functional groups. Pearson correlation with a two-tailed test was applied to analyze the correlation between bud banks density and above-ground plant community composition at different degradation gradients. SPSS 22.0 was used for the analysis of variance and *post-hoc* testing, and Origin pro 9.1 was used for mapping. All tests were based on a two-tailed test of type III sum of squares, and were considered significant at the level of α = 0.05.

## Result

### Response of bud density of different plant functional groups to degradation gradient

**Figure 2:**
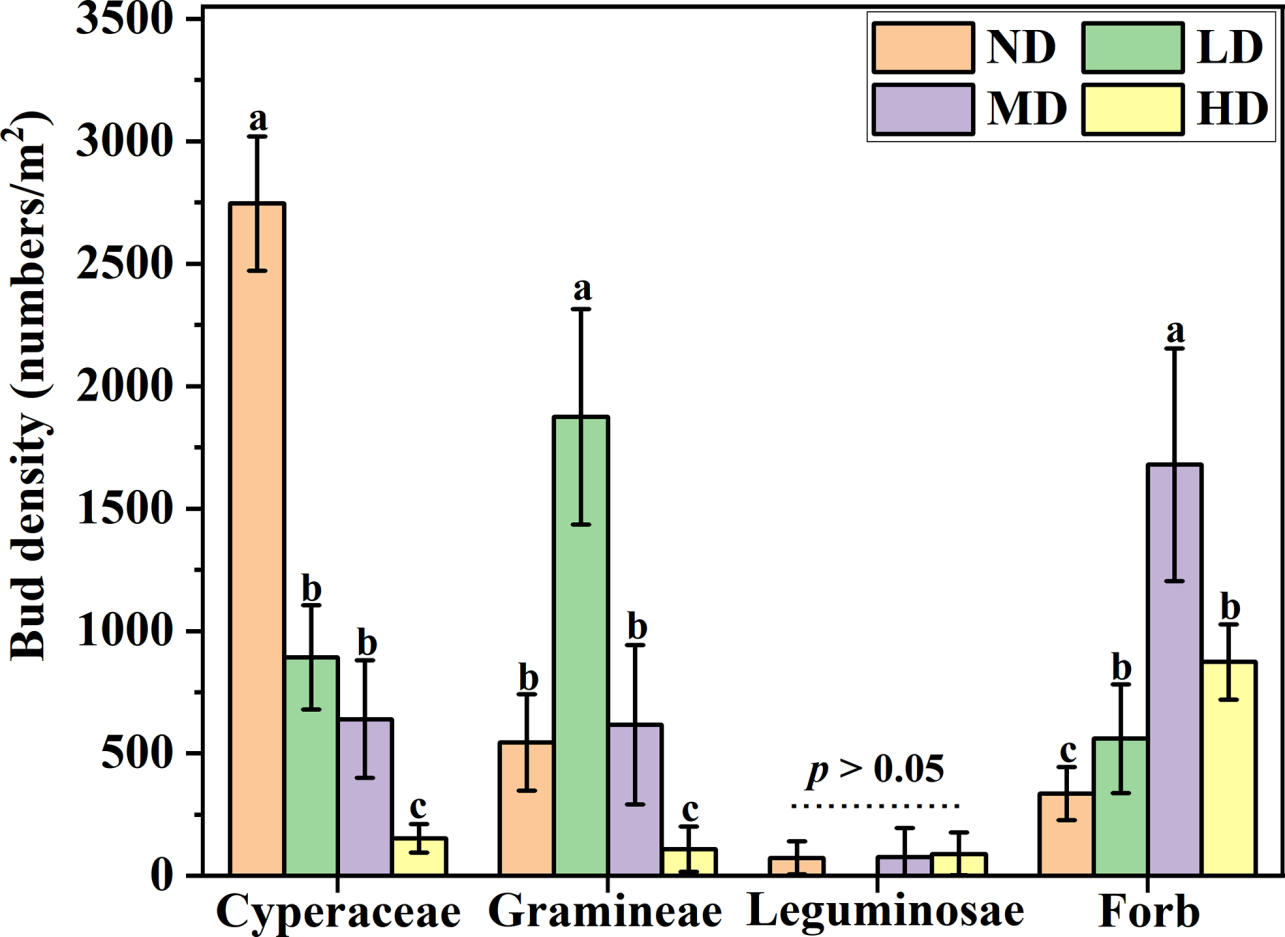
Bud bank density of different plant functional groups under degradation gradient. ND, non-degraded; LD, lightly degraded; MD, moderately degraded; HD, heavily degraded. Different lowercase letters indicate that bud bank density is significantly different among different functional groups P < 0.05 (mean ± standard error; lowercase letters: *P* < 0.05).

Overall, The density of functional group buds showed different responses with the degradation gradient. The density of cyperaceae buds decreases as the degradation gradient increases, and the bud density of cyperaceae on the ND gradient was significantly higher than that of the other three degraded gradient plots (*P* < 0.05), However, there was no significant difference in the density of cyperaceae buds under LD and MD gradients, but both were significantly higher than the cyperaceae bud density of HD gradients (*P* < 0.05). The density of gramineae buds dominates the gradient of LD (60%), while the bud density of forb dominates the gradient of MD (49%). The density of leguminosae buds was not significant under each degradation gradient (*P* > 0.05) (**Figure 2**).

### Response of total bud bank density and bud bank type to degradation gradient

**Figure 3:**
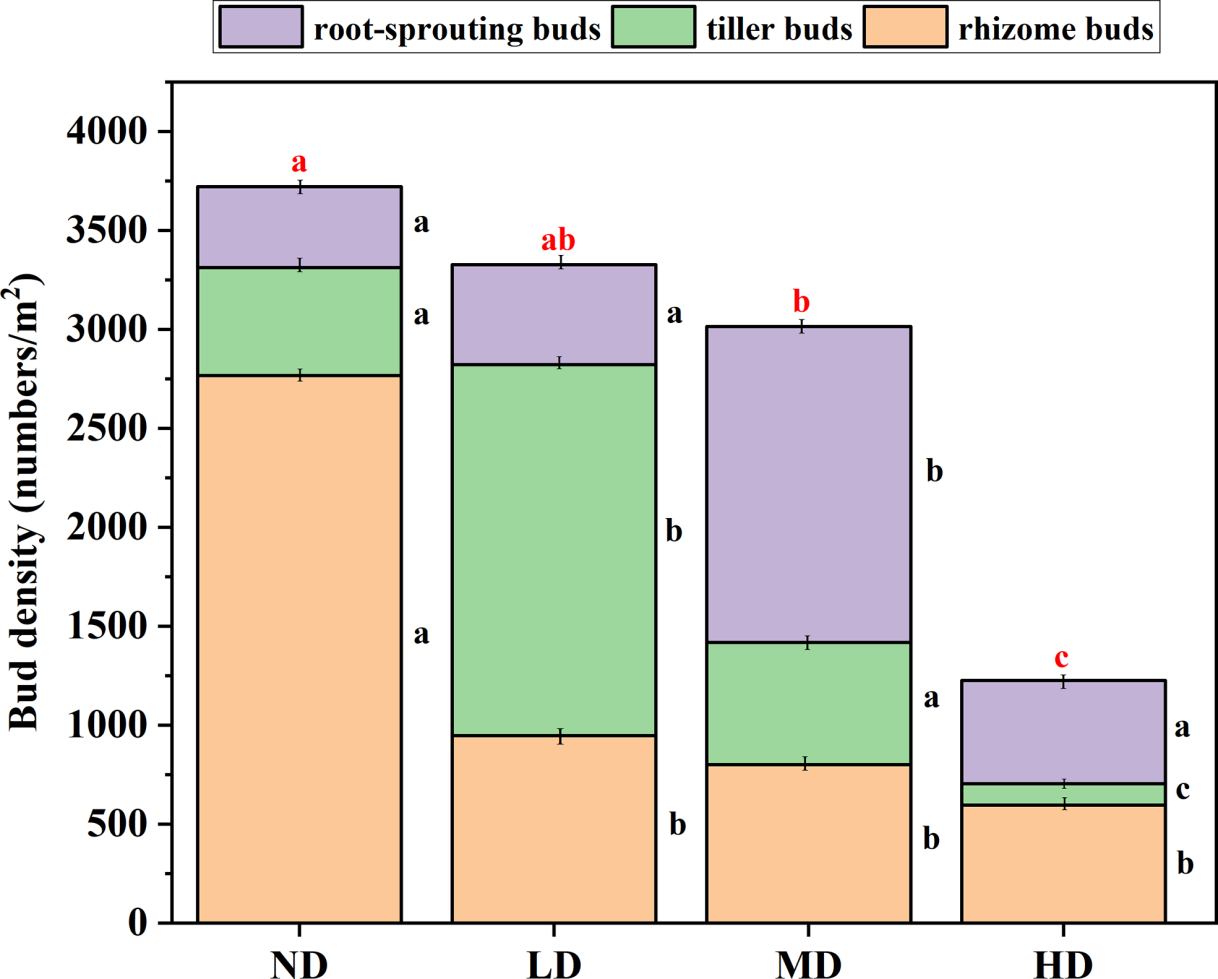
Bud bank density and composition under the degradation gradient of four alpine meadows. ND, non-degraded; LD, lightly degraded; MD, moderately degraded; HD, heavily degraded. Different red lowercase letters indicate significant differences among degradation gradients (*P* < 0.05); Different black lowercase letters indicate significant differences within the degradation gradient (*P* < 0.05), Data are means ± standard error.

Under different degradation gradients, the total bud bank density was different. Specifically, as the degradation gradient intensified, the total bud bank density gradually decreased, the total bud density of the ND gradient (mean ± 1SE: 3699 ± 184 bud/m^2^) was significantly greater than the MD gradient (3014 ± 230 bud/m^2^), and the HD gradient (1126 ± 77 bud/m^2^) (*P* < 0.05) (**Figure 3**), but not significantly different (*P* > 0.05) from that in the LD gradient (3328 ± 294 bud/m^2^). There was no significant difference in bud density between the LD gradient and the MD gradient (*P* > 0.05), but it was significantly higher than the bud density of the HD gradient (172 ± 21%) and (149 ± 21%) (*P* < 0.05).

The density of rhizome buds decreases with the intensification of the degradation gradient, the rhizome buds density of the ND gradient was significantly higher than the LD gradient, the MD gradient, and the HD gradient (*P* < 0.05), However, the difference in rhizome buds density among the last three degradation gradients was not significant (*P* > 0.05). The density of tillering buds dominates under the LD gradient (60%), and under the three degradation gradients, the density of tillering buds decreases with the aggravation of the degradation gradient. Root-sprouting bud density dominates under a MD gradient (53%), which was significantly higher than that under ND, LD, and MD gradients (*P* < 0.05) (**Figure 3**).

### Correlation analysis of bud bank and above-ground plant community composition under different degradation gradients

**Figure 4:**
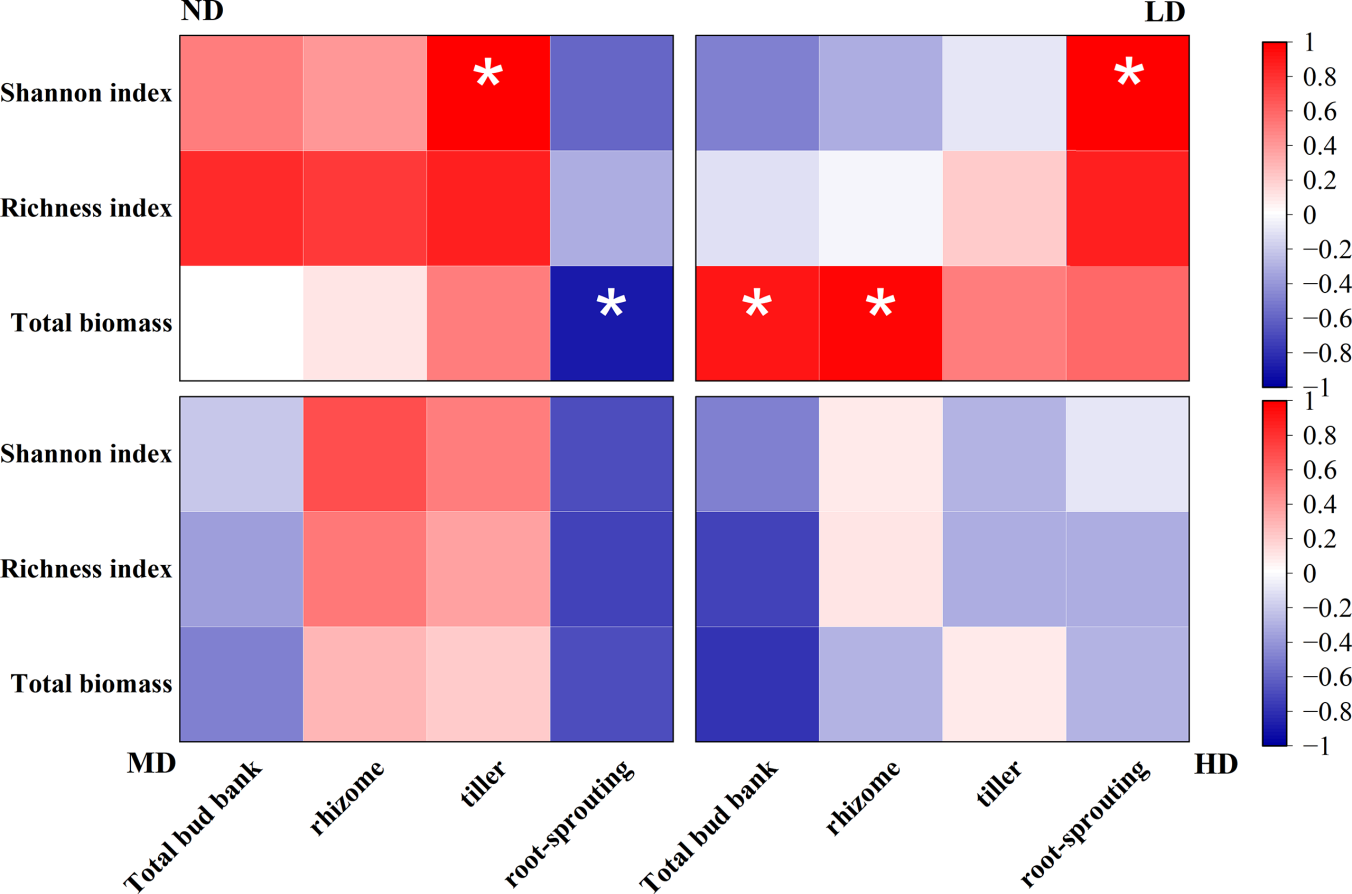
Correlation analysis of bud bank density and above-ground plant community composition under different degradation gradients. The colour of the box indicates the correlation coefficient, red indicates positive correlation, blue indicates negative correlation, the darker the colour, the larger the absolute value of the correlation coefficient. *P < 0.05.

Bud bank density was associated with above-ground plant community composition under different degradation gradients (**Figure 4**). Overall, tiller bud density was significantly positively correlated with the Shannon index of above-ground vegetation under the ND gradient (*P* < 0.05), root-sprouting bud density was significantly negatively correlated with both total above-ground biomass in the ND meadow and the Shannon index in the LD gradient (*P* < 0.05), and total above-ground biomass was significantly positively correlated with total bud bank and rhizome bud density in the LD gradient (*P* < 0.05).

## Discussion

### Analysis of bud bank density renewal mode of plant functional groups under degraded alpine meadow

The ecological role of specific plant functional groups is one of the foundations for understanding vegetation succession (Klimešová and Klimeš, 2007). The types of bud banks among plant functional groups show different survival strategies and adaptive mechanisms to degradation gradients. Specifically, plants with rhizome buds functional groups tend to encroach on resources, which is a competitive expansion strategy, while plants with root-sprouting buds functional groups exhibit evasive behavior in resource utilization, which is a stress-tolerant strategy, the survival strategy of the tiller bud is between rhizome buds and root-sprouting buds, and its survival strategy is the common utilization of resources. In our research, the process of meadow degradation is essentially the suppression of fine herbaceous plants such as cyperaceae and gramineae. With the intensification of degradation, the bud bank density of cyperaceae showed a gradual decrease trend; The density of gramineae bud banks increased from ND to LD meadow, but decreased from LD to HD meadow, indicating that the inhibition effect of grassland degradation on gramineous bud banks depended on the degree of degradation. Grassland degradation is also conducive to the generation of forb bud banks, and the density of bud banks in MD meadow is the highest, this result may be due to the inhibition of fine forage grasses by degradation, which makes forb alleviate the pressure of inter-specific competition and improve the efficiency of resource utilization (Wilkinson, 1999). In all stages of degraded grassland, the response of leguminosae bud density is not sensitive, which is related to the low proportion of leguminosae in the community.

### Influence of degradation gradient on bud bank type and bud bank density

Differences in bud bank implantation position and clonal organ morphology and function lead to differences in the response of different bud bank types to environmental disturbances and degradation gradients (Hendrickson and Briske, 1997; Ott and Hartnett, 2012). Compared with rhizome buds and root-sprouting buds, tiller buds have limited space expansion ability, and they cannot make timely use of distant resources. Generally, tiller buds are located at the base of gramineous plants, the buds are small and wrapped in the leaf axils, protected by leaf sheaths. In contrast, root-sprouting buds are adventitious buds produced by plant roots, which are often used for plant callus inhibition or secondary germination after resource pulses (Biondini et al., 1998; Horvath et al., 2002), so they can partially avoid the negative effects of environmental stress (Klimešová et al., 2017). It is generally believed that rhizome buds have strong branching ability, so that they can maximize resources by increasing the number of branches and shortening the length of spacers under stress conditions (Luo et al., 2014). We found that rhizome buds are dominant in the ND gradient, which may be related to the sensitivity of rhizome buds to water, when the water content in the soil is high, the water will stimulate the expansion of rhizomes and buds and place the ramets in benign patches to maximize resources (Combroux and Bornette, 2004). Tiller buds dominate the LD gradient, due to the combined effects of climate and human activities, the dense and compact grass mat layer in the alpine meadow is destroyed (fragmented) or even completely lost, and the soil nutrient and water retention function gradually weakened, the dominant species of the plant community gradually changed, and *Kobresia pygmaea* in the ND meadow was gradually replaced by *Stipa purpurea*, which prefers dry habitats, so that the density of tiller buds was LD to the highest. Compared with rhizome buds and tiller buds, root-sprouting buds are more adapted to heavily disturbed habitats. Our results show that the density of root-sprouting buds in the MD gradient occupies a dominant position, this stage is an obvious transitional stage, many plants in the above-ground vegetation still have strong asexual reproduction ability and produce more clonal buds, and most of the forb can produce a considerable amount of germinated seeds, which will facilitate the propagation of more forb, so the density of root-sprouting buds is the highest in MD gradient.

In this study, total bud bank density decreased with increasing degradation. From the perspective of degraded succession, the ND and LD gradient belong to the early stage of plant succession, at this time, the dominant plants of the two degraded gradients are cyperaceae (ND) and gramineae (LD), the community mainly relies on clonal organ reproduction and under-ground bud bank renewal, with the highest total bud bank density. By the middle of the succession (MD), most of the forb that can produce seeds appeared in the community, the proportion of sexual reproduction was greatly increased, and the proportion of clonal reproduction decreased (Maliková et al., 2012). On the other hand, from the perspective of forage edibility and grazing value, due to the more poisonous weed seeds and buds in the soil at this stage (forb bud bank density accounts for about 60%, while good forage buds sink density accounts for about 40%), which makes the replacement of species deviate from the normal trajectory, and the recovery ability of fine grasses such as cyperaceae and gramineae is greatly reduced, so the total bud bank density is reduced. At the later stage of succession (HD), the land is nutrient-poor, the turf layer is fragmented, and the degree of degradation exceeds the ecological restoration threshold, at this stage, the species is relatively poor, the community structure is relatively simple, and the total bud bank density is the lowest.

### Dynamic analysis of bud bank density and above-ground vegetation community composition

In grassland ecosystems, below-ground bud banks represent the adaptive capacity and reproductive potential of plants and are indicative in predicting the process and direction of grassland succession. Many studies have shown that bud bank density is closely related to species diversity, grassland productivity and community stability, a high number of bud banks can improve the structure and function of grassland ecosystems (Chen et al., 2015; Chen et al., 2018). In our study, tiller bud density was significantly and positively correlated with the Shannon index under the ND gradient, root-sprouting bud density was significantly correlated with the Shannon index under the LD gradient, and changes in rhizome bud density were not significantly different from above-ground species diversity. This implies that in ND meadows, due to the large proportion of cyperaceae plants such as *Kobresia pygmaea*, their dominance suppresses the growth of other species in the community and reduces the diversity of the plant community, while with the small number of gramineae in the community, the increase in tiller bud density will increase the species diversity of the above-ground community under the ND gradient. Similarly, in LD meadows, where the degradation gradient is further intensified, leguminosae and forb plants gradually appear in the community, and both have an increased proportion of root-sprouting buds contributing to the bud bank, expanding ecological niches and gaining more space and resources through rapid growth, and as the density of newly expanded root-sprouting buds increases, their corresponding above-ground plant species diversity also increases significantly. Species diversity and above-ground biomass in MD and HD was not significantly correlated with bud bank density, which was related to the mode of reproduction under the two degradation gradients.

In previous studies it was found that in alpine meadow ecosystems, population reproduction is dominated by asexually reproducing perennials, which contribute more than 90% (Wang X. et al., 2020), but the soil seed bank is still a non-negligible part of the population, except that compared to asexual reproduction, sexual reproduction produces seedlings that are less resistant and less likely to survive under alpine environmental conditions (Amiaud and Touzard, 2004). In the MD and HD stages, the growth of cyperaceae and gramineae is affected and their dominance is lost, the bare leaking plots provide favourable conditions for the growth of other plants (especially forb) and the increased proportion of the seed bank enriches species diversity to some extent (**Table 1**). We further infer that during the succession of alpine meadow plant communities, there is no consistency between the soil bud bank and the composition of the corresponding above-ground vegetation, specifically, the density of the bud bank at the beginning of the succession (ND, LD) is positively correlated with species diversity, while the density of the bud bank at the middle and late succession (MD, HD) is not correlated with species diversity, and the value of the contribution of the bud bank to above-ground vegetation gradually decreases as the gradient of degradation increases.

## Conclusions

The different bud bank types have different adaptation strategies to the degradation gradient: at the beginning of succession (ND, LD), the bud bank types are dominated by rhizome and tiller buds respectively, at the middle of succession (MD), the bud bank types are dominated by root-sprouting buds, while at the end of succession (HD), the habitat is poor and the structurally simple community cannot recover to its original stage by its own regulation. The density of soil bud banks at the beginning of succession (ND, LD) was positively correlated with species diversity, while the density of bud banks at the middle and late succession (MD, HD) was not correlated with species diversity, and the value of the contribution of bud banks to above-ground vegetation gradually decreased as the degradation gradient increased. In addition, the degradation of the MD gradient was more conducive to species diversity in terms of above-ground plant community composition.

## Data availability statement

The original contributions presented in the study are included in the article/supplementary material. Further inquiries can be directed to the corresponding author.

## Author contributions

JY wrote the manuscript and executed the technical assays and statistical analysis. JY and X-tW designed the experiment and edited the manuscript text. MZ contributed to data collection and interpretation. All authors contributed to the article and approved the submitted version.

## Funding

This study was supported by the Natural Science Foundation of Tibet Autonomous Region (XZ202101ZR0114G); Fund Project for Central and Local Universities in 2022 (KY2022ZY-01), and the National Natural Science Foundation of China (42161012).

## Acknowledgments

We sincerely thank Liang-feng Liu, Xin-wei Liu, Pei-jun Ju, Xuhui Chen, Chuan Zhao, Shuo-lei Huang for their valuable comments on the pictures of the article. We also wish to send our profound gratitude to reviewers for their invaluable inputs during the review process.

## Conflict of interest

The authors declare that the research was conducted in the absence of any commercial or financial relationships that could be construed as a potential conflict of interest.

## Publisher’s note

All claims expressed in this article are solely those of the authors and do not necessarily represent those of their affiliated organizations, or those of the publisher, the editors and the reviewers. Any product that may be evaluated in this article, or claim that may be made by its manufacturer, is not guaranteed or endorsed by the publisher.
